# Practical Notes and Formulæ

**Published:** 1887-06

**Authors:** 


### PRACTICAL NOTES AND FORMULÆ.

**For Cholera Morbus, Etc.**—We are indebted to Dr. A. C. Davidson for the following excellent formula useful to arrest vomiting, and especially recommended in the vomiting and purging of cholera morbus:

℞. Alcohol

Carbolic acid ... aaʒ v,

Chloroform ... ʒ vi.

Dose—10 to 20 drops in a wine glass of water.

**Cannabis Indica in Acute and Chronic Dysentery.**—Mr. Rennie first tried the officinal tincture in doses of 20 minims three times daily, but he has since found that this dose sometimes produced slight toxic symptoms, and he has consequently reduced the quantity to 15 minims, and prescribes it according to the following formula:

℞. Tinct. cannabis indicæ ... *m* xv,
Bismuth, subcarb ... gr. v,
Mucilage, acac ... ʒ ss,

Misce, et adde

Tinct. zingiberis ... *m* xx,
Tinct. carda. co ... *m* xx,
Spt. chloroform ... *m* xx,
Aqua cinnamom, ad ... ℥ i

Ter die sum.

Even in these doses it is necessary sometimes to order it to be taken after meals, as it occasionally produces vertigo. It is pleasant to take, and there is no subsequent nausea. The administration requires to be kept up for several days after all symptoms have ceased.—*Therapeutic Gazette.*

**To Stop Toothache.**—Gesell-Fells makes the following mixture, which is an oily liquid, and introduced in the tooth cavity has proved very effective:

℞. Camphor ... gr. lxxv,
Chloral hydrati ... gr. lxxv,
Cocaini muriat ... gr. xv.

**Prescription for Headache.**—The following is from Dujardin-Beaumetz:

Ethoxycafeine ... gr. xii,

Sodii salicylat ... gr. xv,

Aquæ destill., ad ... ℥ i,

Dose, teaspoonful or tablespoonful.

**Guibaut** recommends the following in psoriasis:

℞. Sodii arsen..............................gr. 1-60,
Ext. gentian..............................gr. iss,

In pill form.—Two or three pills after each meal. Also frictions twice daily with—

Adipis.............................℥ iii,

Acip. pyrogallic..............................ʒ iiss to ʒ iv.

Also a thorough cleansing with soap every two days.—*Les Nouveaux*, No. 23.

**Rheumatism.**—A writer in *American Medical Journal* recommends as the best rheumatic remedy the following:

℞. Fl. ext. black cohosh..............................℥ j,

Fluid ext. prickly ash bark..............................℥ j,

Syrup simplex..............................℥ viij.

Mix. Teaspoonful three times per day; or, if you wish, you can give larger doses, and oftener. You may add to this, iodide potash, ℥ ss. Give podophyllin, or any other good "liver medicine," and you may safely tell your patient that if he will continue that medicine for one week, he will be able to attend to business.

**Ear Ache.**—

℞. Muriate of morphia..............................gr. v,
Sulphate of atropia..............................gr. j,
Oleum olivæ..............................ʒ j,
Neutral glycerin..............................ʒ jss.

M. Sig. Drop from 3 to 5 drops into the ear, and repeat every hour until relieved from pain, taking care to plug the ear with cotton after applying the medicine.—*Medical Specialist.*

**A Laxative and Tonic Wine.**—

℞. Tincture calisaya..............................
Tincture simaruba..............................
Tincture gentian..............................
Tincture bitter orange peel..............................aa f. ʒ ijss,
Tincture ignatia bean..............................ʒ ss,
Sherry wine enough to make..............................O ij.

Mix and filter. The wine is tonic, carminative and laxative. The dose is from 1 to 2 fluid ounces.—*Prog. Medical.*

**Cocaine Cotton**—Is prepared, according to *Rundschau* (Prag), by saturating 100 parts cotton, deprived of fat, with 100 parts of a 3-per cent. aqueous solution of cocaine. Used for toothache.

Cocaine-Morphine cotton is prepared by saturating 100 parts cotton, deprived of fat, with 100 parts 3-per cent. solution of cocaine (aqueous) in which 2-per cent. morphine has been dissolved. Used for tooth and earache.

Cocaine-Boric acid cotton. This is made by saturating 100 parts of a solution prepared as follows:

℞. Cocaine .................................... 2 parts,
Acidi carbolici.................................. 1 part,
Acidi borici...... ........................... 2 parts,
Glycerini .......................................... 4 parts,
Aquæ .................. ................ 100 parts.

Ft. solut.

Used as a pain-killer and for burns.—*Druggist.*

**Acute Dysentery.**—Dr. Stowers in *Medical World* says my treatment for the above mentioned disease is simply this. I first order:

℞. Sodæ bitartratis................................

Sodæ et potassæ tart..........................aa ℥ ss

M. S.—Dissolve in a half a glass of water, and give every two hours until the character of the action is changed.

I also order flannel cloths to be wrung out of as warm water as the patient can bear, and applied to the abdomen every fifteen minutes.

As soon as the character of the actions has changed, I order:

℞. Bismuth subnit..................................
Pulv. ipecacuanhæ comp........................aa gr. x.

M. S.—Take one every three or four hours, as may be required to control the actions of the bowels; and keep the patient quiet.

I find milk to be the best food, but order a light diet at all times. I have read a great deal on this subject and believe this to be as good as, if not superior to, any treatment of this affection.

**Chills.**—Pof. Walker combines capsicum with quinine in intermittent fever. He recommends the following prescription:

℞. Quininæ sulph....... ..............................℥ j,

Oleoresinæ capsici...............................gr. iij.

M. et in pil. no. xxx div.
Sig.—Three after meals.

He continues these large doses until he has prevented chills for two days. He then puts patient on two-thirds the dose until eight days after the last paroxysm. For the next seven days two pills bedtime.—*Medical World.*

**Cocaine in the Treatment of Dysentery.**—Dr. J. E. Winters (*New York Medical Journal*, April 24, 1886) has used from 10 to 20 drops of an eight-per-cent. solution of cocaine in a drachm of water as a rectal injection repeated every two, four, six, or eight hours, according to symptoms, and with the most satisfactory results.—*Epitome.*

